# First isolation of West Nile virus in Brazil

**DOI:** 10.1590/0074-02760180332

**Published:** 2019-01-17

**Authors:** Lívia Caricio Martins, Eliana Vieira Pinto da Silva, Livia Medeiros Neves Casseb, Sandro Patroca da Silva, Ana Cecília Ribeiro Cruz, Jamilla Augusta de Sousa Pantoja, Daniele Barbosa de Almeida Medeiros, Arnaldo Jorge Martins, Ermelinda do Rosário Moutinho da Cruz, Marialva Tereza Ferreira de Araújo, Jedson Ferreira Cardoso, Marcos Antônio Correia Rodrigues da Cunha, Gilton Luiz Almada, Alessandro Pecego Martins Romano, Maria Guadalupe Dias Pestana Santos, Gilsa Aparecida Pimenta Rodrigues, Jannifer Oliveira Chiang, Juarez Antonio Simões Quaresma, Valéria Lima Carvalho, Pedro Fernando da Costa Vasconcelos

**Affiliations:** 1 Instituto Evandro Chagas, Seção de Arbovirologia e Febres Hemorrágicas, Ananindeua, PA, Brasil; 2 Instituto Evandro Chagas, Seção de Patologia, Ananindeua, PA, Brasil; 3 Instituto Evandro Chagas, Centro de Inovação tecnológica, Ananindeua, PA, Brasil; 4 Secretaria de Estado da Saúde do Espírito Santo, Vitória, ES, Brasil; 5 Ministério da Saúde, Secretaria de Vigilância em Saúde, Brasília, DF, Brasil; 6 Secretaria de Saúde do Município de Venécia, Venécia, ES, Brasil

**Keywords:** West Nile virus, horses, Brazil

## Abstract

**BACKGROUND:**

Serological evidence of West Nile virus (WNV) infection has been reported in different regions of Brazil from equine and human hosts but the virus had never been isolated in the country.

**OBJECTIVES:**

We sought to identify the viral etiology of equine encephalitis in Espírito Santo state.

**METHODS:**

We performed viral culture in C6/36 cells, molecular detection of WNV genome, histopathology and immunohistochemistry from horse cerebral tissue. We also carried out sequencing, phylogenetic analysis and molecular clock.

**FINDINGS:**

Histopathologic analysis from horse cerebral tissue showed injury related to encephalitis and WNV infection was confirmed by immunohistochemistry. The virus was detected by reverse transcription quantitative polymerase chain reaction (RT-qPCR) from brain tissue and subsequently isolated in C6/36 cells. WNV full-length genome was sequenced showing the isolated strain belongs to lineage 1a. The molecular clock indicated that Brazilian WNV strain share the same common ancestor that were circulating in US during 2002-2005.

**MAIN CONCLUSIONS:**

Here we report the first isolation of WNV in Brazil from a horse with neurologic disease, which was clustered into lineage 1a with others US WNV strains isolated in beginning of 2000’s decade.

West Nile virus (WNV) is a member of the Japanese Encephalitis serocomplex within *Flaviviridae* family and *Flavivirus* genus.^1^ Originally, WNV was isolated from a human with fever in Uganda, 1937.^2^ Later it would be detected in the Middle East, France, South Asia and Australia. In 1999, the virus was introduced into USA and since then, it has spread from Canada to Argentina.^3^

WNV is maintained in nature by transmission between *Culex* mosquitoes, in particular *Cx. pipiens*, considered its principal vector, and certain passerine birds, its main reservoir host.^4^ Horses and humans are incidental or “dead-end” hosts, since they develop low level viremia which is insufficient to contribute to WNV spread via mosquitoes.^4^ ^,^ ^5^

WNV is a neuropathogen to human, equine, and many other mammalian and avian species.^4^ In humans, around 80% of infected people are asymptomatic and 20% will develop West Nile Fever (WNF) which can range from mild acute febrile illness, to neurological diseases including meningitis, encephalitis, and acute flaccid paralysis.^6^ Regarding horses, when they develop disease the main symptoms are fever, depression, loss of appetite, colic, encephalitis with ataxia, limb weakness, recumbency and muscle fasciculation.^7^

Phylogenetic analysis established nine WNV lineages according to geographic location and the type of host. Lineages 1 and 2 are associated with the majority of human outbreaks in Europe, Africa, Middle East, Americas and Oceania. Lineages 3, 4, 6, and 9 are from Europe. Lineage 5 is from India. Lineages 7 and 8 are from Africa.^1^

In South America, WNV was isolated for the first time in Argentina from horses in 2006 and two years later, the virus was isolated in Colombia from captive flamingoes.^8^ ^,^ ^9^ Regarding Brazil, after the spread of the virus in North America, in 2003, the National WNV Surveillance System was created, based on the recommendations of Pan American Health Organization (PAHO) and World Health Organization (WHO). Since then, any suspected human or animal case of WNF is investigated following the established protocol and biological samples are referred for differential laboratory diagnosis.

Over the years, some studies reported serological evidence of WNV in Brazil from different hosts and regions indicating the circulation of the virus,^10^ ^,^ ^11^ ^,^ ^12^ ^,^ ^13^ but the virus had never been isolated in the country. We describe the first isolation of WNV in Brazil, obtained from a horse with encephalitis in Espírito Santo state.

## MATERIALS AND METHODS

*Clinical information* - An adult male horse from Pedra Grande region on the São Mateus municipality, Espírito Santo state, Brazil, with signs of colic developed neurological manifestations on the 25th of April 2018 including dysphagia, ataxia in anterior limbs, muscle tremors, shaking, opisthotonos, lateral decubitus, intense sweating, pedaling movements and other alterations indicating hemineglect. Within 24 hours, the animal showed signs of paralysis of the pelvic limbs, difficulty chewing, and not responding to needle stimulation along the spine. The animal was euthanised and specimens were submitted to the Evandro Chagas Institute, National Reference Laboratory for Arbovirus, for laboratory diagnosis, including testing for WNV. The sample was assigned registration number BE AN 854747.

Before the disease episode, the animal had been immunised with Tri-Equi-Hertape vaccine covering protection against Eastern equine encephalitis virus, Western equine encephalitis virus, equine influenza viruses (types A1 and A2, including Kentucky 92) and tetanus toxoid.

*Virus isolation* - Approximately 0.05 gram of central nervous system (CNS) sample was homogenised in 1.0 mL phosphate-buffered saline, pH 7.4, containing 5% fetal bovine serum with penicillin (100 U/mL), streptomycin (100 mg/mL) and gentamicin (0.05 mg/mL), using one 5 mm stainless steel bead in a TissueLyser (Qiagen, Hilden, Germany) set to 26 Hz for four min. The sample was frozen at -80ºC overnight, then thawed and centrifuged at 16,266 g for 10 min (4ºC) and then further clarified with a 0.22-µm nylon syringe filter. Then 100 µL of the supernatant filtrate was inoculated into C6/36 cells and after 1 h of adsorption at 28ºC, 1.5 mL of Leibovitz’s L-15 maintenance medium (supplemented with 2% Foetal Bovine Serum, 0.1% Penicillin 100 U/mL and Streptomycin 100 mg/mL, 10% Tryptose Phosphate Broth and 1% Non-essential amino acids) was added into 20 cm^2^ tissue culture tube. The cells were incubated at 28ºC and observed during seven days, scraped from the tubes and collected on slides to perform indirect immunofluorescence assay (IFA) using hyperimmune mouse ascitic fluids to *Flavivirus* group-reactive (including antibodies to the following viruses: Bussuquara, Ilhéus, Rocio, Cacipacoré, Dengue 1, 2, 3, 4, Naranjal, Zika and West Nile-chimera), WNV, as well as, to *Alphavirus* group-reactive and Oropouche virus to rule out infection by other families of viruses.

*Histopathology and immunohistochemistry* - Paraffin-embedded tissue samples were processed for histopathology and stained with hematoxylin and eosin (HE).^14^ For immunohistochemistry, an adapted Streptavidin Alkaline Phosphatase assay^15^ with anti-WNV polyclonal antibody serum was used.

*Detection of WNV genome* - The RNA was extracted from CNS tissues supernatant by using Maxwell 16 Tissue LEV Total RNA Purification Kit (Promega) using Maxwell automated system. Reverse transcription quantitative polymerase chain reaction (RT-qPCR) for detection of WNV genome was performed following established protocol.^16^ The assay was performed on a 7500 Real Time PCR System (Applied Biosystems) using Superscript III Platinum One-Step qRT-PCR System kit (Invitrogen). We also performed RT-qPCR for detection of Saint Louis encephalitis virus, for differential laboratory diagnosis, as described.^17^

*Sequencing* - The cell culture supernatant was extracted with QIAamp Viral RNA Mini Kit. The metagenome sequencing process started with the ssRNA. The synthesis of the first and second strand was performed using SuperScript VILO MasterMix and NEBNext Second Strand Synthesis Module, respectively. The reaction was purified with PureLink PCR Purification Kit, following manufacturer’s instructions.

The cDNA library was prepared and sequencing using the methodology described in the Nextera XT DNA Library Preparation Kit on a Miniseq (Illumina, Inc).

The genome sequence was determined using the *De Novo Assembler* methodology in IDBA-UD program^18^ and SPAdes.^19^ The inspection, annotations of putative open reading frames (ORF) genes and additional analysis were performed using the Geneious v.9.1.6 software (Biomatters, New Zealand). All contigs were aligned and compared against the database of virus proteins available in NCBI through the Diamond.^20^

*Phylogenetic and evolutionary analysis* - Initially, a multiple sequencing alignment, using the entire ORFs of the Brazilian strain and more than 1,500 WNV nucleotide sequences available on NCBI, was performed using Mafft v7.310 software^21^ and for visual inspection we used Geneious v.9.1.8 software. Subsequently, recombination events were evaluated using the Phi-test implemented on SplitsTree4 v4.14.6 program.^22^ The best-fitting model of nucleotide substitution was determined using jModelTest v.2.1.10.^23^ A maximum likelihood (ML) tree was constructed using the RAxML v.8.2.11 software.^24^ Afterward, a new nucleotide dataset was selected to perform evolutionary analysis based on the full phylogenetic tree using the entire ORFs of 166 WNV strains comprising other South American WNV strains and basal lineages to the cluster including the Brazilian WNV strain.

The temporal structures for each one and the combined WNV clades were verified by using the TempEst v1.5.1, and all data were plotted using ggplot2 with the R programming language. To estimate the time to the most recent common ancestor (MRCA) for each monophyletic clusters and the rate of nucleotide substitutions per site, we used the Markov Chain Monte Carlo (MCMC) employing Bayesian approach available on the BEAUTI and BEAST v.1.10.1 package and BEAGLE library. However, we firstly established the prior MCMC chosen by testing all models and determining Bayes factors (log10 BF > 3), as well as, the best clock and coalescent models were selected using the log marginal likelihood estimation (MLE) under path sampling (PS) and stepping-stone sampling (SS) methods implemented in BEAST. The best model used was the general time-reversible with invariant sites (GTR + I + T4) (Supplementary data II).

We calculated different coalescent models based parametric Constant and non-parametric was the Skygrid and Skyline models, as well as, the molecular clock models using the strict molecular clock (Strict) and the uncorrelated relaxed lognormal molecular clock (UCLN). The MCMC method was employed with three independently runs each composed by 100 million generations and uncertainties of the parameters estimates were excluded after assessing the initial 10% of burn-in by calculating the Effective Sample Size (ESS) within a confidence interval of 95% Highest Probability Density (HPD) value, using the TRACER v.1.7.1.^25^ A maximum clade credibility tree (MCC) was produced and annotated by the use of TreeAnnotator in the BEASTusing FigTree v.1.4.3.^26^

## RESULTS

**Fig. 1:**
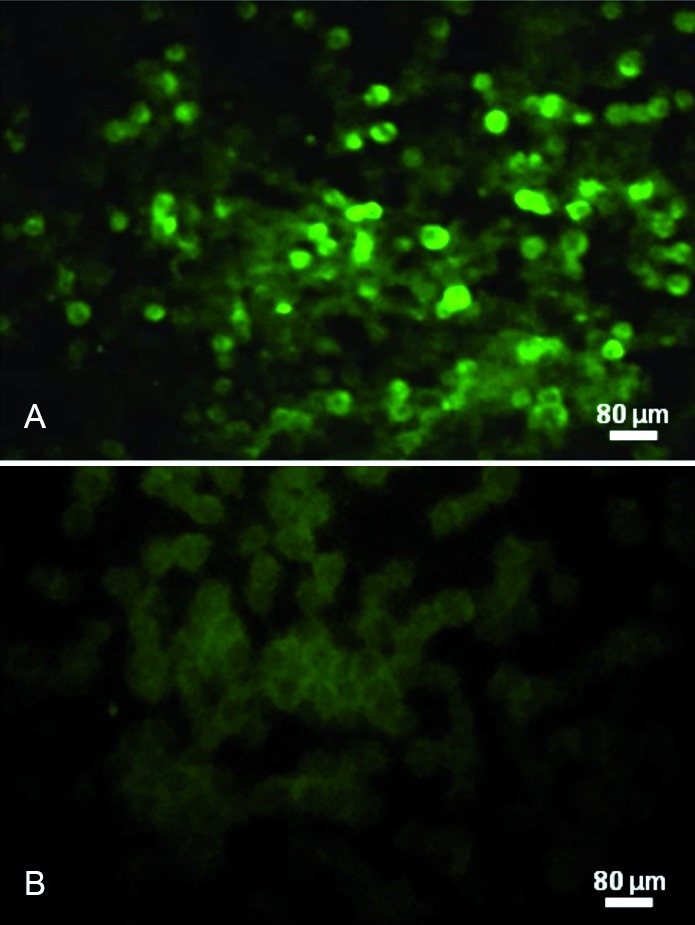
detection of West Nile virus (WNV) antigen in C6/36 cells by indirect immunofluorescence assay (IFA). (A) WNV antigen (green) in infected cells detected by IFA using flavivirus group-reactive hyperimmune mouse ascitic fluid. (B) Uninfected cell control by IFA using the same hyperimmune mouse ascitic fluid.

*Virus isolation and detection of WNV genome* - C6/36 cells inoculated with the sample BE AN 854747 (CNS material) presented cytopathic effect (CPE) in the fourth day post-inoculation (dpi) characterised by cell death and formation of syncytia. On the seventh dpi, the indirect immunofluorescence assay showed approximately 75% positive cells for both *Flavivirus* and WNV polyclonal antibodies (Fig. 1). The horse’s brain samples were also positive to WNV by RT-qPCR (Ct: 31.2).

**Fig. 2:**
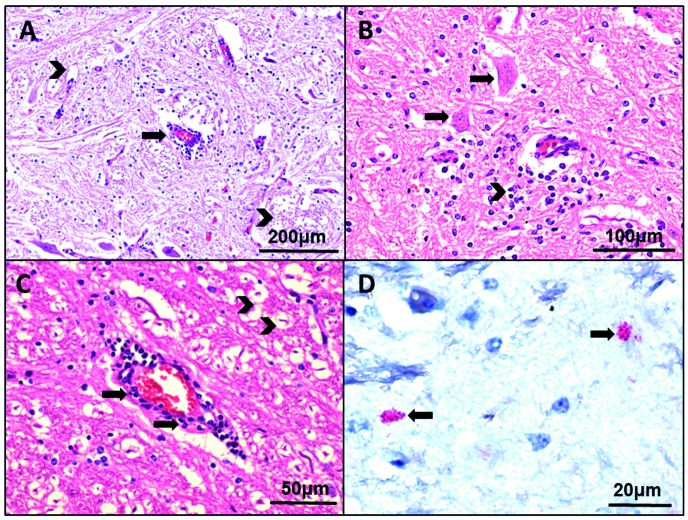
photomicrograph of cerebral tissue of a horse with neurologic manifestation from Espírito Santo state with description of histopathological aspects and positive immunostaining for West Nile virus (WNV). (A) Inflammatory infiltrate predominantly constituted by mononuclear cells with perivascular cuffing (arrow) and neuronal necrosis (arrow heads). (B) Brain tissue showed neuronophagy (arrows) and associated gliosis (arrow head). (C) Perivascular infiltrate with congestion (arrows) and foci of neuronal necrosis of different intensities (arrow heads). (D) Immunohistochemistry of WNV antigen (reddish staining) in the cell cytoplasm in several foci of brain tissue (arrows).

*Histopathology and immunohistochemistry* - The histological sections of the horse’s brain showed tissue alterations characterised by inflammatory infiltration of the parenchyma, which is sometimes distributed around the vessels and consist of mononuclear and polymorphonuclear leukocytes, area of variable neuronal necrosis, neuronophagia, microglial nodules and gliosis. Anti-WNV antibodies represented by deposition of reddish granules in the cytoplasm of the cells showed the presence of viral antigens in the cerebral parenchyma (Fig. 2).

**Fig. 3:**
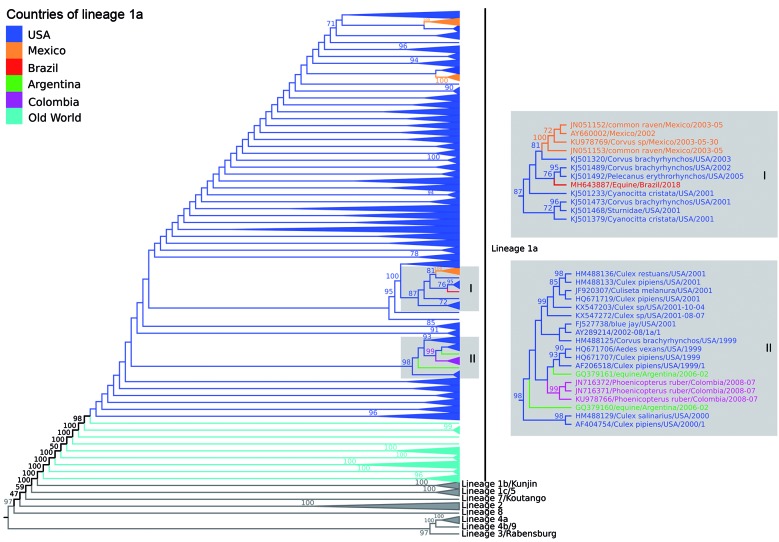
midpoint phylogenetic tree of nucleotide sequences using only polyprotein coding region of 1582 West Nile virus (WNV) strains representing different viral lineages. The analysis of those nucleotide sequences was performed using the maximum likelihood method based on the GTR matrix-based model. Different phylogenetic groups are assigned to previously defined lineages; highlighted in dark blue, strains included in lineage 1a from USA and in light blue strains included in lineage 1a from Europe, Asia and Africa. Strains from Brazil (red), Mexico (orange), Colombia (purple), and Argentina (green) were also identifies on the phylogenetic tree. Numbers over each main node of the tree correspond to bootstrap values (1000 replicates). Values < 70 are not supported by reproducible topologies. The bar represents nucleotide substitutions along of the branch.

*Phylogenetic and evolutionary analysis* - The ML phylogenetic trees, using GTR+I+T4 model, showed similar topology identifying all WNV lineages previously described. Our data showed that the BE AN 854747 strain (GenBank accession number: MH643887) belonged to lineage 1a related to others strains from United States and Mexico (Fig. 3; Supplementary data I). The Brazilian strain was more closely related with two US strains isolated from *Corvus brachyrhynchos* (KJ501489) and *Pelecanus erythrorhynchos* (KJ5011492) in 2002 and 2005, respectively, with average of identity of 99.0% (nucleotide) and 99,6% (amino acid).

**Fig. 4:**
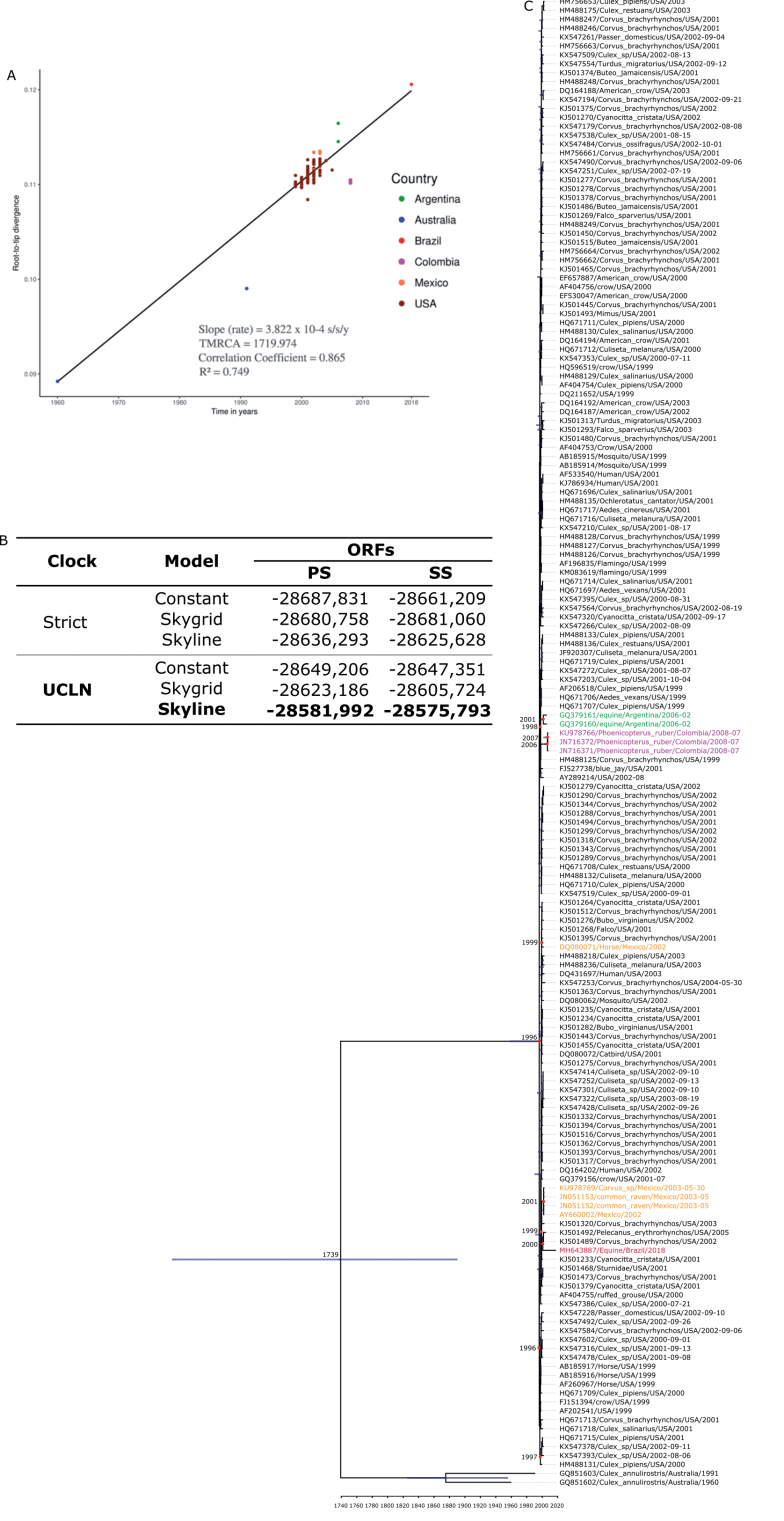
(A) Posterior probability densities and root-to-tip analysis based on maximum likelihood tree (Supplementary data II) demonstrated a good temporal correlation (R^2^ = 0749), indicating that evolutionary clock models were appropriate for inferring the evolutionary origins of the West Nile virus (WNV) samples. It was used, with a relaxed uncorrelated log-normal molecular clock and a Bayesian skyline tree model. (B) Log marginal likelihood estimates, using distinct molecular clock and coalescent model combinations for temporal reconstruction using entire WNV open reading frames (ORFs) by path sampling model selection (PS) and stepping-stone model selection (SS) methods. The best-fitting model is highlighted in boldface. Coalescent models used were the parametric Constant models and the non-parametric was the Skygrid and Skyline models. The molecular clock models used were the strict molecular clock (Strict) and the uncorrelated relaxed lognormal molecular clock (UCLN). (C) Maximum clade credibility (MCC) tree of WNV genomes constructed using BEAST version 1.10.1 software. Colors of branches indicate geographic locations per the color key. Branch lengths correspond to lengths of time.

To build molecular clock tree, we checked recombination to lineages 1a and 1b. The Phi-test did not find statistical significant evidence for recombination (p = 0.1979). Furthermore, 166 WNV strain, including the Brazilian strain were previously selected and a linear regression analysis was performed (Fig. 4A) based on ML tree (Supplementary data II), showing good temporal signal for this dataset.

The best molecular clock and coalescent model were uncorrelated relaxed lognormal molecular clock (UCLN) and Skyline models, respectively (Fig. 4B). The evolutionary analysis of the 166 WNV strains isolated from 1960 to 2018 indicates that the most recent common ancestor of all South America strains were from US and probably were introduced by independent events in Argentina, Brazil and Colombia where the estimated time were 16.5 years ago (15.79 - 17.39, 95% HPD), 17.2 years ago (16.34 - 18.06, 95% HPD) and 11.2 years ago (10.64 - 11.76, 95% HPD), suggesting that the ancestors of these clusters were circulating in 2002, 2000 and 2007, respectively. The introduction of Brazilian strain was estimated by most closely related sequences of USA KJ501492/2005 and KJ501489/2002 (Fig. 4C).

## DISCUSSION

We report the first isolate and detection of WNV in Brazil from an equine CNS sample collected in a rural area of Espírito Santo state. The virus caused histopathologic alterations in the cerebral tissue of the horse compatible with encephalitis and similar to those previously described for WNV.^6^ Infected C6/36 cells presented characteristic morphologic changes commonly observed for flaviviruses. The phylogenetic analysis of the full-length genome showed that this WNV isolate is included in lineage 1a, which comprises strains isolated from Europe, Africa, and the Americas.^1^ We also identified greater genetic relatedness of the BE AN 854747 isolate with North American strains as well as Central (Mexico) and South America strains (Colombia and Argentina).^27^ ^,^ ^8^ ^,^ ^9^

The molecular clock suggested that Brazilian WNV share the same ancestor with US strains that were circulating between 2000 to 2005 and probably was introduced in Brazil from a different event than Argentine and Colombian. We observed that Mexico presented multiple introductions all of them related to US strains and the oldest Mexican strains, introduced in 2001, are most closely to the Brazilian strain (BE AN 854747).

Since 1999 when WNV emerged in USA, the virus has spread throughout the Americas from Canada to Argentina causing several thousand cases of neurological disease, including cases of fatalities in humans, but with higher rates of mortality in birds and horses. After the detection of the virus in the Americas, many efforts have been made to detect the possible circulation of the virus in Brazil. Thus, neutralising antibodies were first detected in equines and chickens in Pantanal region in 2009 and 2010;^10^ ^,^ ^11^ and later in equines in the Northeast region and again in the Pantanal.^12^ In 2015, the country reported the first human case of WNV encephalitis with flaccid paralysis in Piauí state in which all CDC confirmation criteria for WNF were filled including the detection of neutralising antibodies against the virus.^13^ This indicates that WNV had been previously enzootically circulating but never had been isolated in the country. It is worth pointing out that in Brazil there is the co-circulation of other flaviviruses like Dengue, Zika, Yellow Fever, Saint Louis encephalitis, Ilheus and others, which complicates the serological diagnosis of these viruses, due to the extensive flavivirus cross-reactivity in serological assays.^28^ Furthermore, this cross-reactivity can lead to cross-protection which may prevent WNV from causing large epidemics in humans in Brazil. However, WNV may become an important pathogen to animals, especially wild life and animals of production, affecting directly the economy of the country. Currently the health, agricultural and environmental authorities are investigating this equine case and implications for local human and animal populations. At the national level, the Brazilian surveillance system investigates neuroinvasive arboviral disease, creating notification systems for both human and animal surveillance, as well as a guide to clinical management and establishment of sentinel hospitals to investigate neurologic cases in each state of the country.^29^ Nevertheless, it is important that with the first isolation of WNV, the Brazilian surveillance systems be further alert to human and equine neurological cases compatible with WN Fever. The notification and investigation of neurological diseases in equidae as well as the epizootic events of wild birds should be improved as they may be the key to the detection of WNV before outbreaks of large magnitude.

*Ethics* **-** Biological samples of animals were obtained and sent by Epidemiologic Surveillance, Ministry of Health, to Evandro Chagas Institute, national reference laboratory, to confirm the laboratory diagnosis. The euthanasia of the animals was made by *anesthetic* overdose following parameters established by Guía para la Vigilancia, Detección y Respuesta para las Encefalitis Equinas.^30^ This procedure was carried out for diagnostic purposes, and therefore does not require consideration by ethics committees according to Brazilian law number 11.794 of October 8th, 2008.
