# Supplementary material for: First isolation of West Nile virus in Brazil
Source: Mem Inst Oswaldo Cruz. 2019 Jan 17;114:e180332. doi: 10.1590/0074-02760180332 (PMC6343470; doi:10.1590/0074-02760180332)
Supplement: Supplementary file 1 [file 1678-8060-mioc-114-e180332-s2.pdf]

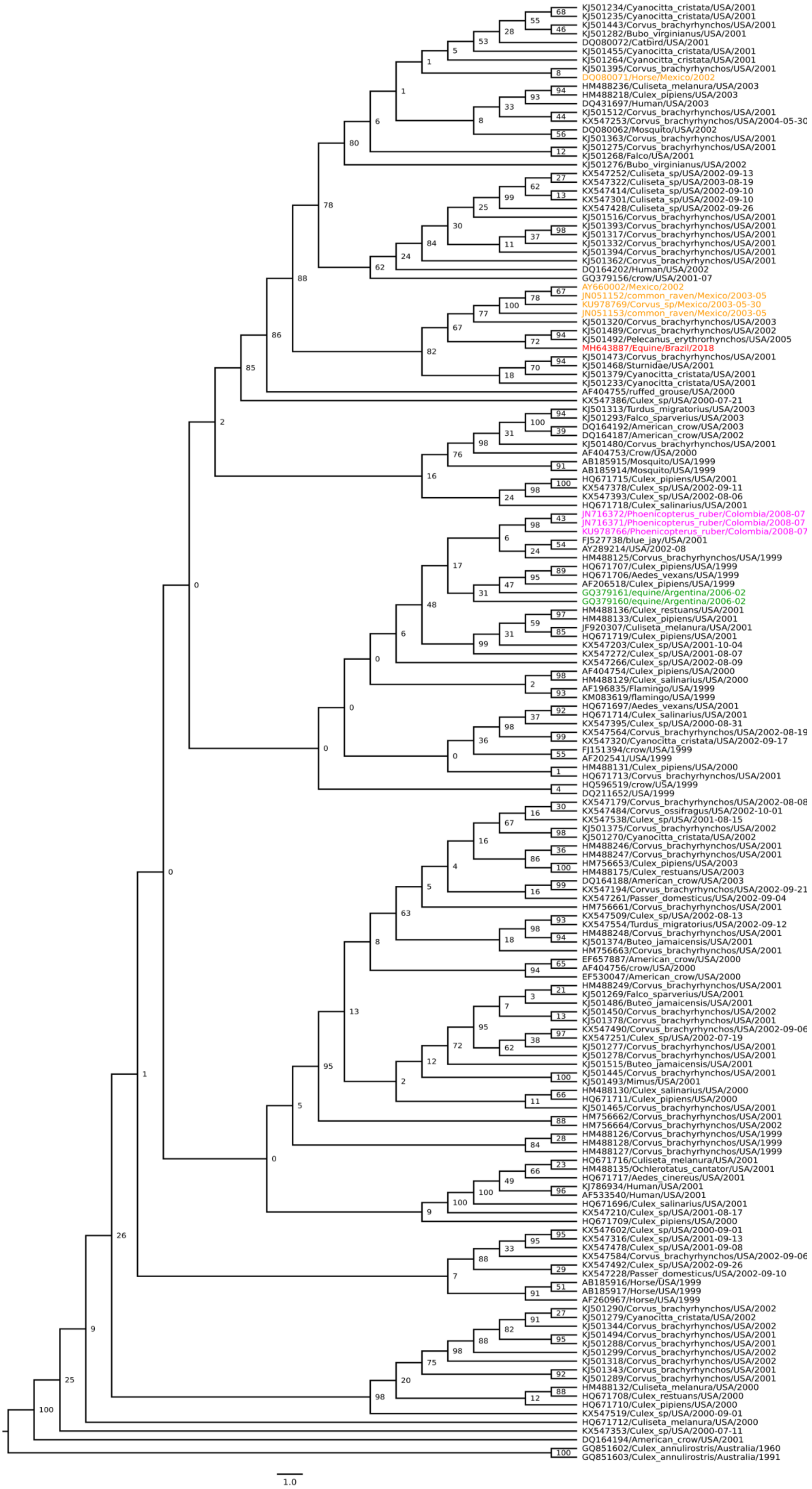

Fig. 2: midpoint maximum likelihood (ML) phylogenetic tree of nucleotide sequences using only polyprotein coding region of 167 West Nile virus (WNV) strains. The analysis of those nucleotide sequences was performed using the ML method based on the GTR+I matrix-based model. Data used to support linear regression analysis on Fig. 4A.

cc BY

REFERENCES

1. Rambaut A, Lam TT, Carvalho LM, Pybus OG. Exploring the temporal structure of heterochronous sequences using TempEst (formerly Path-O-Gen). Virus Evol. 2016; 2(1): vew007.

2. Wickham H. ggplot2 Elegant graphics for data analysis. New York: Springer-Verlag; 2009.

3. Suchard MA, Lemey P, Baele G, Ayres DL, Drummond AJ, Rambaut A. Bayesian phylogenetic and phylodynamic data integration using BEAST 1.10. Virus Evol. 2018; 4(1): vey016.

4. Ayres DL, Darling A, Zwickl DJ, Beerli P, Holder MT, Lewis PO, et al. BEAGLE: an application programming interface and high-performance computing library for statistical phylogenetics. Syst Biol. 2012; 61(1): 170-3.

5. Lartillot N, Philippe H. Marginal likelihood estimation: computing bayes factors using thermodynamic integration. Syst Biol. 2006; 55(2): 195-207.

6. Xie W, Lewis PO, Fan Y, Kuo L, Chen MH. Improving marginal likelihood estimation for Bayesian phylogenetic model selection. Syst Biol. 2011; 60(2):
