# Supplementary material for: First isolation of West Nile virus in Brazil
Source: Mem Inst Oswaldo Cruz. 2019 Jan 17;114:e180332. doi: 10.1590/0074-02760180332 (PMC6343470; doi:10.1590/0074-02760180332)
Supplement: Supplementary file 2 [file 1678-8060-mioc-114-e180332-s1.pdf]

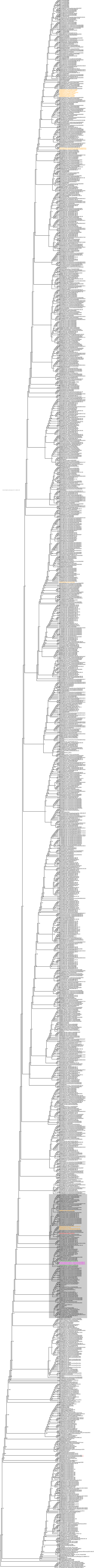

Fig. 1: extended maximum likelihood (ML) phylogenetic tree from Fig. 3. Midpoint phylogenetic tree of nucleotide sequences using only polyprotein coding region of 1582 West Nile virus (WNV) strains representing different viral lineages. The analysis of those nucleotide sequences was performed using the ML method based on the GTR matrix-based model. Gray box highlight strains more closer related to Brazilian strain used to perform the molecular clock analysis including and two taxon from lineage 1b choose as outgroup (*).
